# Labor Market Integration of High-Skilled Immigrants in Canada: Employment Patterns of International Medical Graduates in Alternative Jobs

**DOI:** 10.3390/healthcare10091705

**Published:** 2022-09-06

**Authors:** Tanvir C. Turin, Nashit Chowdhury, Deidre Lake, Mohammad Z. I. Chowdhury

**Affiliations:** 1 Department of Family Medicine, Cumming School of Medicine, University of Calgary, Calgary, AB T2N 4N1, Canada; 2 Department of Community Health Sciences, Cumming School of Medicine, University of Calgary, Calgary, AB T2N 4N1, Canada; 3 Alberta International Medical Graduates Association, Calgary, AB T2E 3K8, Canada

**Keywords:** immigrants, job market integration, international medical graduates, decision making, Canada

## Abstract

**Background:** International medical graduates (IMGs) in Canada are individuals who received their medical education and training outside Canada. They undergo a complex licensing procedure in their host country and compete for limited opportunities available to become practicing physicians. Many of them cannot succeed or do not have the resources or interest to undergo this complex and unpredictable career pathway and seek alternative career options. In this study, we aimed to understand how IMGs integrate into the alternative job market, their demographic characteristics, and the types of jobs they undertake after moving to Canada. **Methods:** An anonymous cross-sectional, online, nationwide, and open survey was conducted among IMGs in Canada. In addition to demographic information, the questionnaire included information on employment status, types of jobs, professional experience, and level of medical education and practice (e.g., specialties, subspecialties, etc.). We conducted a survey of 1740 IMGs in total; however, we excluded responses from those IMGs who are currently working in a clinical setting, thus limiting the number of responses to 1497. **Results:** Of the respondents, 43.19% were employed and 56.81% were unemployed. Employed participants were more likely to be older males, have stayed longer in Canada, and had more senior-level job experience before moving to Canada. We also observed that the more years that had passed after graduation, the higher the likelihood of being employed. The majority of the IMGs were employed in health-related nonregulated jobs (50.45%). The results were consistent across other demographic characteristics, including different provinces, countries of origin, gender, time since graduation, and length of stay in Canada. **Conclusions:** This study found that certain groups of IMGs, such as young females, recent immigrants, recent graduates, and less experienced IMGs had a higher likelihood of being unemployed. These findings will inform policymakers, immigrant and professional service organizations, and researchers working for human resources and professional integration of skilled migrants to develop programs and improve policies to facilitate the employment of IMGs through alternative careers.

## 1. Background

Internationally trained health professionals are a part of a high-skilled global migration trend that has been occurring in relatively higher volume during the last few decades [1,2]. Among these professionals, international medical graduates (IMGs), who are educated and trained as physicians in their home countries [3,4], are one of the groups who struggle the most in terms of professional integration in the host countries. Professionals in this category are particularly vulnerable within the host country’s labor market, as they face several challenges when they attempt to enter into professional physician practice [2]. This has created a large pool of IMGs seeking professional integration in Canada, but the system has not kept pace with the regulatory infrastructure to integrate them [5]. Although there are some differences between provinces in the licensure process of physicians, generally, all are required to provide credentials for verification of their education, demonstrate language proficiency in English or French, and successfully complete professional licensure exams. IMGs also compete for a limited number of residency positions to complete their licensure process; thus, many remain unable to practice medicine even after passing all of the required exams [6].

It has been reported that IMGs generally seldom considered seeking any career other than becoming a physician [7], and this usually leads to delay in seeking any other career [8]. This delay can deprive IMGs of time that may be required to prepare for any new career and obtain any required related work training or experience to allow for success in a decent alternative career [9,10]. Most studies on IMGs only focused on the areas of work [11,12] and experience of the licensed IMGs [13] and barriers and struggles [14] to licensing for the unlicensed IMGs. A survey of 283 migrant physicians in Sweden found that before becoming licensed physicians, 61% of the respondents had, on average, worked for 20 months in Swedish healthcare in various alternative jobs [15]. Examples included nurses and assistant nurses, caretakers, medical assistants, administrators, interpreters, researchers, or teachers of courses closely connected to healthcare. No further detail on the distribution of those employments or their relation with sociodemographic traits were reported in that study or other studies on unlicensed IMGs in Canada [16] or other IMG-accepting countries (e.g., the US, the UK, Australia, etc.). Against this backdrop, different types of jobs are sought by IMGs as a way of getting into the job market or developing a long-term career. Given the lack of data and discussion around job market integration of IMGs, little is known about this issue, especially concerning unlicensed IMGs.

To better understand the landscape of job market integration patterns of IMGs in Canada, we conducted an anonymous online survey of IMGs in Canada who are not in any type of physician’s license-related job. We aimed to describe their demographic characteristics and employment status and explore the type of jobs they have undertaken after migration to Canada.

## 2. Methods

### 2.1. Survey Population

The survey was intended for all IMGs residing in Canada who had the status of permanent resident or had Canadian citizenship. IMGs residing in other countries were not included. Due to the time frame of the educational journey to become a medical graduate, the IMGs were >18 years of age.

### 2.2. Survey Questionnaire

The anonymous, voluntary, and open survey consisted of questions developed to capture information on participant demographics, current job status, and job type if they were employed. The survey was created based on the existing literature and our work in recent years, particularly with regard to alternative career pathways for IMGs. Most of the questions were close-ended. Across the 13 screen pages, the median and average number of question items per screen page was 3 and 3.3, respectively. There were adaptive questions in the questionnaire so that respondents could jump past inapplicable questions based on their answers. No personal information was collected during the survey. We pre-tested the survey for clarity, relevance of questions, and survey duration with IMG members of the research team. The survey had a landing page describing the survey information, and consent was implied when participants clicked the “Go to Survey” button from this page. We also provisioned a $10 gift card to randomly selected 50 participants who completed the survey. The survey was web-based and developed using Qualtrics survey software (Qualtrics, Provo, UT, USA) [17], which provided the option to prevent duplicate submissions from a single Internet Protocol (IP) address. But no IP-address-related information was retrieved from the survey software as a data point.

### 2.3. Survey Dissemination

Adopting a convenience sampling approach [18], we reached out to a number of organizations in Canada who provide support for IMGs in their professional resettlement and requested to disseminate the survey through their electronic mailing list. The email consisted of an information poster that included a link to the anonymous online survey. One month after the first contact, a reminder request was sent to all potential organizations. We also disseminated the survey via social media in regular intervals. The study received approval from the University of Calgary’s Conjoint Health Research Ethics Board.

### 2.4. Data Analysis

Descriptive analysis was used to calculate frequencies and percentages. As all the variables were categorical variables, we used χ^2^ tests or Fisher’s exact tests in order to compare the various groups. During our analysis, we checked if the data met the assumptions of the χ^2^-test (the data in the cells are frequencies of counts rather than any transformed form, the levels or categories of the variables are mutually exclusive, each subject contributes to only one cell, study groups are independent, and the expected cell frequencies are 5 or more in at least 80% of the cells, as well as no cell has an expected frequency of less than one) [19]. When the expected cell frequency did not meet the threshold, we used Fisher’s exact test [20], and corresponding *p*-values are reported. We also used logistic regressions [21] to examine both unadjusted and multivariable-adjusted relationships between employment status and IMGs’ sociodemographic factors (age, gender, current living location, country of origin, country of medical graduation, length of stay in Canada, years passed after graduation, and highest level of practice before coming to Canada). Statistical analyses were performed using STATA version 15.1 (StataCorp, College Station, TX, USA). Supplementary Table S1 contains the checklist for reporting results of Internet e-surveys (CHERRIES) [22].

## 3. Results

In total, 1740 participants responded to the survey during the period of November 2019–June 2020. On average, participants completed 85.92% (SD 29.15) of the questionnaire, with 1149 (76.75%) participants completing the survey in its entirety. Incomplete questionnaires with missing responses were also included in the analysis. For this analysis, we included 1306 respondents after excluding the IMGs who were working as partial or fully licensed physicians.

Regarding employment status, 564 (43.19%) respondents were employed and 742 (56.81%) were unemployed. Table 1 shows the demographics of the IMG participants across the two groups: employed and unemployed. Among the younger participants of age ≤ 29, higher unemployment (73.6%) was observed. Among females, higher proportion (60.53%) of unemployment was observed. Among relatively newcomers to Canada, unemployment was higher (72.66%). Unemployment was higher among relatively recent immigrant IMGs (81.90%). Among relatively recent graduates, higher unemployment (81.90%) was observed, whereas higher (52.20%) employment was observed in participants whose graduation was >15 years ago.

Table 2 shows the unadjusted and multivariable-adjusted odds ratios (OR) and 95% confidence intervals (CI) associated with the likelihood of being employed. The likelihood of being unemployed was significantly associated with the female gender (*p* = 0.015) in the multivariable-adjusted model. The likelihood of employment was significantly associated with a longer stay in Canada (*p* = 0.001 for 3–5 years or 0.003 for >15 years) and increased number of years since graduation (*p* = 0.006 for 3–5 years or 0.001 for >15 years). Women were 30% less likely to be employed. In comparison to the participants who had been in Canada for <3 years, the odds of employment for participants living in Canada for 3–5 years was 1.85, for 6–10 years was 2.90, for 11–15 years was 3.29, and for >15 years was 2.31. We observed a dose–response relationship between the numbers of years passed after graduation and the likelihood of being employed, with the OR being the highest for respondents with >15 years (OR 5.28; 95% CI 2.04–13.67).

Among the 564 respondents who were employed, job-type information was available for 551 respondents. Table 3 shows the different types of jobs in which the IMGs reported being employed. The majority of them reported being involved in health-related nonregulated jobs (278, 50.45%). For both males and females, a higher number of participants reported being involved in health-related nonregulated jobs (male, 56.18% and female, 41.48%). Being employed in health-related nonregulated jobs, compared to other job-types, was the highest across all age groups (62.50% for ≤29 year, 51.52% for 30–39 years, 46.63% for 40–49 years, and 46.43% for ≥50 years). A similar pattern of job type was observed across the groups for time since graduation and length of stay in Canada.

## 4. Discussion

We conducted a Canada-wide survey among IMGs to explore the employment pattern of IMGs. We found that while the majority of the respondents were unemployed (43.08% vs. 56.92%), the proportion of unemployment was higher among the younger age group. A potential reason might be that younger IMGs might be more focused on completing their licensing exams and applications and thus chose not to work immediately after immigration. Additionally, the issue that it may be relatively difficult for someone to find a job during the early period after immigrating to a new country could also contribute to these findings. We also observed that female IMGs were less likely to be employed, which could relate to immigrant women choosing to prioritize the responsibilities of taking care of the family at home. Similar results have been observed in other studies [23,24]. The explanation provided for this type of phenomenon is based on a family investment model [25,26], where married immigrant women tend to work as secondary workers in the family to invest in their husbands’ careers and fulfill family responsibilities. Alternatively, there have been reports that refute this phenomenon and postulate that skill assimilation of educated immigrant women occurs over time and results in their increasing integration into the job market over time [27]. In this regard, we also observed that the number of years passed after graduation was also associated with the likelihood of being employed. Gaining more experience in the years following graduation corresponds with our finding that previous experience in senior-level jobs was more related to being employed.

Some findings of this study, such as having more experience in their country of origin and being male, were associated with employment in alternative careers and may contradict previous understanding of the situation. It has been reported that many employers are not aware of IMGs’ prior knowledge and experience and others believe they are overqualified due to their vast experience, which purportedly impedes their employment [28,29]. In addition, although it has been reported that females tend to show more interest in alternative careers [5], we found males to be more likely to be employed in alternative careers.

We found that the majority of employed participants entered either regulated or nonregulated health-related careers. It has been conceptualized that as IMGs are only trained to work as a physician, they do not know how to work in business or administrative environments, hence they tend to find employment in fields that are related to health [14]. The higher proportion of IMGs in nonregulated health-related alternative careers found in our study can be explained by comments from participants in another study. In that study, they found that IMGs encounter many systemic barriers in terms of finding a job in regulated health sectors due to the expense and complexities associated with the licensing process of those jobs [14].

Our study has several limitations typical of open-survey and convenient-sample-based studies, especially those where the denominator is not available. Through our extensive efforts to have the survey reach IMGs across Canada, we had a large sample that responded to the survey. However, that large sample did not provide generalizability of our findings. It should also be noted that there was no available centralized contact list of IMGs in Canada that we could have used to conduct our survey. Additionally, the data were collected through an online survey. As there was an incentive of $10 CAD as a draw prize for the participants, false entries were a possibility. The survey was designed to prevent resubmission from the same IP link, thus preventing multiple submissions by the same person; however, there is often a workaround for those who are overly enthusiastic. Second, for the classification of employment category, the participants may not have interpreted regulated versus nonregulated jobs properly (especially those who are in nonregulated jobs), despite providing a clear definition of regulated jobs in the questionnaire. We believe the effect of such misclassification was very minimal and did not affect our findings. Another limitation of this study is that we did not differentiate employment between full-time and part-time positions. Many IMGs may prefer part-time jobs to make time to study for their licensure procedure or may not seek a job for the same reason. We cannot determine if the rate of unemployment was due to the barriers of unemployment or due to a lack of interest among IMGs to be employed. The strength of our survey is that we were able to include diverse participants from most of the parts of Canada. The questionnaire was developed by the Principal Investigator (TCT) and NC, who were IMGs in alternative careers themselves, and was reviewed by the knowledge users (IMGs and DL, an IMG-related organization lead), thus ensuring the appropriateness of the questions and involving knowledge users in the research process.

### Community Engaged Research Approach

We have conducted this study as a part of our community-engaged (IMG and associated community) program of research. We have research team members who are themselves IMG community members (TCT and NC). We also have been closely partnering [30] with an IMG service providing organization (Alberta International Medical Graduates Association, DL) for this study. Activities and knowledge products related to this study have always been taken to the community members for their feedback as well as their learning and capacity building [31] through continued community engagement [32].

## 5. Conclusions

In this study, we explored the employment pattern of IMGs in alternative careers and found that there is a higher rate of unemployment among certain groups such as females, younger people, recent immigrants, and less experienced IMGs. Organizations, policymakers, and researchers working on facilitating professional integration of IMGs in Canada can use these findings to develop strategies to help IMGs obtain employment in alternative careers. Specific focus needs to be provided on developing systematic career counselling models and tools [33], especially because IMGs generally never got training and exposure of jobs beyond being a physician. This dilemma can be approached through providing proper information support, developing skill-building platforms, creating networking opportunities, and facilitating transferable skill enhancement. Additionally, efforts of removing individual and systemic barriers need to be done through different stakeholder engagement, including potential employers and policy makers. As this is the first comprehensive study on this topic, the insights gained from the results are likely to serve as baseline information to guide future research and development of interventional programs for facilitation of IMGs’ employment through alternative careers.

## Figures and Tables

**Table 1:** Characteristics of the survey respondent IMGs.

Variables	Current Working Position		*p*-Value *
	Employed, *n* (%)	Unemployed, *n* (%)	
*N*	564 (43.19)	742 (56.81)	
*Age group (years)*			
≤29	66 (26.40)	184 (73.60)	<0.001
30–39	273 (46.11)	319 (53.89)	
40–49	164 (49.85)	165 (50.15)	
≥50	57 (44.19)	72 (55.81)	
*Gender*			
Female	345 (39.47)	529 (60.53)	<0.001
Male	210 (50.48)	206 (49.52)	
Other	3 (50.00)	3 (50.00)	
*Province (currently living)*			
Alberta	203 (48.68)	214 (51.32)	<0.001
British Columbia	46 (38.66)	73 (61.34)	
Manitoba	7 (23.33)	23 (76.67)	
New Brunswick	3 (37.50)	5 (62.50)	
Newfoundland and Labrador	25 (83.33)	5 (16.67)	
Nova Scotia	9 (50.00)	9 (50.00)	
Nunavut	0 (0.00)	1 (100.00)	
Ontario	204 (37.71)	337 (62.29)	
Prince Edward Island	0 (0.00)	2 (0.28)	
Quebec	26 (40.00)	39 (60.00)	
Saskatchewan	16 (57.14}	12 (42.86)	
*Country/region of origin*			
East Asia and Pacific	34 (65.38)	18 (34.62)	<0.001
Europe and Central Asia	28 (53.85)	24 (46.15)	
North America, Latin America, and the Caribbean	64 (50.79)	62 (49.21)	
Middle East and North Africa	110 (34.16)	212 (65.84)	
South Asia	202 (39.22)	313 (60.78)	
Sub-Saharan Africa	91 (47.89)	99 (52.11)	
*Country/region where medical graduation was obtained*			
East Asia and Pacific	43 (62.32)	26 (37.68)	<0.001
Europe and Central Asia	43 (41.75)	60 (58.25)	
North America, Latin America, and the Caribbean	62 (53.45)	54 (46.55)	
Middle East and North Africa	111 (35.02)	206 (64.98)	
South Asia	183 (38.53)	292 (61.47)	
Sub-Saharan Africa	80 (46.51)	92 (53.49)	
*Length of stay in Canada (years)*			
<3	108 (27.34)	287 (72.66)	<0.001
3–5	126 (42.86)	168 (57.14)	
6–10	178 (52.51)	161 (47.49)	
11–15	53 (56.38)	41 (43.62)	
>15	75 (56.39)	58 (43.61)	
*Years passed after graduation*			
<3	19 (18.10)	86 (81.90)	<0.001
3–5	57 (34.34)	109 (65.66)	
6–10	127 (41.50)	179 (58.50)	
11–15	111 (44.22)	140 (55.78)	
>15	237 (52.20)	217 (47.80)	
*Highest level of practice before coming to Canada*			
Medical student	62 (33.16)	125 (66.84)	0.012
Resident	82 (39.81)	124 (60.19)	
Attending physician/hospital physician	77 (46.11)	90 (53.89)	
Consultant physician/specialist	126 (42.00)	174 (58.00)	
General practitioner/family physician	201 (48.55)	213 (51.45)	
Department head	12 (50.00)	12 (50.00)	

Note: Due to missing responses, some variable cell counts may not add up to total population number. * *p*-value from Pearson’s chi-squared test or Fisher’s exact test as appropriate.

**Table 2:** Factors associated with being employed among the survey respondent IMGs.

Variables	Current Working Position (Unemployed vs. Employed)	
	Unadjusted OR * (95% CI ^†^)	Adjusted OR (95% CI)
*Age group (years)*		
≤29	Reference	Reference
30–39	2.39 (1.72–3.30)	0.94 (0.56–1.59)
40–49	2.77 (1.94–3.95)	0.74 (0.37–1.48)
≥50	2.21 (1.41–3.45)	0.56 (0.25–1.28)
*Gender*		
Female	0.64 (0.51–0.81)	0.70 (0.53–0.93)
Male	Reference	Reference
Other	0.98 (0.20–4.92)	0.40 (0.02–7.81)
*Province (currently living)*		
Alberta	Reference	Reference
British Columbia	0.66 (0.44–1.01)	0.79 (0.50–1.26)
Manitoba	0.32 (0.13–0.76)	0.39 (0.16–0.97)
New Brunswick	0.63 (0.15–2.68)	0.40 (0.07–2.21)
Newfoundland and Labrador	5.27 (1.98–14.03)	1
Nova Scotia	1.05 (0.41–2.71)	1.40 (0.47–4.12)
Nunavut	1	1
Ontario	0.64 (0.49–0.83)	0.85 (0.63–1.15)
Prince Edward Island	1	1
Quebec	0.70 (0.41–1.20)	1.03 (0.55–1.93)
Saskatchewan	1.41 (0.65–3.04)	1.39 (0.61–3.20)
*Country/region of origin*		
East Asia and Pacific	Reference	Reference
Europe and Central Asia	0.62 (0.28–1.36)	1.91 (0.47–7.69)
North America, Latin America, and the Caribbean	0.55 (0.28–1.07)	1.01 (0.29–3.53)
Middle East and North Africa	0.27 (0.15–0.51)	0.81 (0.20–3.18)
South Asia	0.34 (0.19–0.62)	1.11 (0.35–3.48)
Sub-Saharan Africa	0.49 (0.26–0.92)	1.39 (0.33–5.84)
*Country/region where medical graduation was obtained*		
East Asia and Pacific	Reference	Reference
Europe and Central Asia	0.43 (0.23–0.81)	0.51 (0.16–1.62)
North America, Latin America, and the Caribbean	0.69 (0.38–1.28)	0.97 (0.31–3.01)
Middle East and North Africa	0.33 (0.19–0.56)	0.39 (0.11–1.39)
South Asia	0.38 (0.23–0.64)	0.38 (0.14–1.03)
Sub-Saharan Africa	0.53 (0.30–0.93)	0.53 (0.14–2.04)
*Length of stay in Canada (years)*		
<3	Reference	Reference
3–5	1.99 (1.45–2.74)	1.85 (1.29–2.66)
6–10	2.94 (2.16–3.99)	2.90 (1.98–4.26)
11–15	3.44 (2.16–5.46)	3.29 (1.88–5.77)
>15	3.44 (2.29–5.17)	2.31 (1.33–4.01)
*Years passed after graduation*		
<3	Reference	Reference
3–5	2.37 (1.31–4.28)	2.77 (1.34–5.74)
6–10	3.21 (1.86–5.55)	3.22 (1.41–7.35)
11–15	3.59 (2.06–6.26)	3.45 (1.44–8.23)
>15	4.94 (2.91–8.40)	5.28 (2.04–13.67)
*Highest level of practice before coming to Canada*		
Medical student	Reference	Reference
Resident	1.33 (0.88–2.01)	1.24 (0.72–2.14)
Attending physician/hospital physician	1.72 (1.12–2.65)	1.40 (0.79–2.47)
Consultant physician/specialist	1.46 (0.99–2.14)	0.86 (0.48–1.54)
General practitioner/family physician	1.90 (1.33–2.73)	1.36 (0.81–2.28)
Department head	2.02 (0.86–4.75)	1.18 (0.39–3.53)

Note: * OR = odds ratio; ^†^ CI = confidence intervals.

**Table 3:** Types of jobs in which IMGs reported working.

Variables	Health-Related (Regulated Alternative Career), *n* (%)	Health-Related (Non-Regulated Alternative Career), *n* (%)	Non-Health-Related (Professional Job), *n* (%)	Non-Health-Related (Non-Professional Job), *n* (%)	*p*-Value *
*N*	101 (18.33)	278 (50.45)	67 (12.16)	105 (19.06)	
*Age group (years)*					
≤29	2 (3.13)	40 (62.50)	2 (3.13)	20 (31.25)	<0.001
30–39	45 (17.05)	136 (51.52)	44 (16.67)	39 (14.77)	
40–49	36 (22.09)	76 (46.63)	14 (8.59)	37 (22.70)	
≥50	18 (32.14)	26 (46.43)	4 (7.14)	8 (14.29)	
*Gender*					
Female	72 (21.18)	191 (56.18)	22 (6.47)	55 (16.18)	<0.001
Male	28 (13.86)	84 (41.58)	42 (20.79)	48 (23.76)	
Other	0 (0.00)	0 (0.00)	2 (66.67)	1 (33.33)	
*Province (currently living)*					
Alberta	34 (17.00)	105 (52.50)	17 (8.50)	44 (22.00)	<0.001
British Columbia	9 (19.57)	19 (41.30)	4 (8.70)	14 (30.43)	
Manitoba	1 (16.67)	3 (50.00)	0 (0.00)	2 (33.33)	
New Brunswick	1 (33.33)	2 (66.67)	0 (0.00)	0 (0.00)	
Newfoundland and Labrador	0 (0.00)	0 (0.00)	25 (100.00)	0 (0.00)	
Nova Scotia	2 (22.22)	4 (44.44)	1 (11.11)	2 (22.22)	
Nunavut					
Ontario	45 (22.28)	114 (56.44)	13 (6.44)	30 (14.85)	
Prince Edward Island					
Quebec	3 (11.54)	17 (65.38)	0 (0.00)	6 (23.08)	
Saskatchewan	4 (26.67)	6 (40.00)	1 (6.67)	4 (26.67)	
*Country/region of origin*					
East Asia and Pacific	12 (36.36)	17 (51.52)	1 (3.03)	3 (9.09)	0.050
Europe and Central Asia	5 (18.52)	15 (55.56)	2 (7.41)	5 (18.52)	
North America, Latin America, and the Caribbean	11 (18.03)	36 (59.02)	4 (6.56)	10 (16.39)	
Middle East and North Africa	18 (16.51)	61 (55.96)	6 (5.50)	24 (22.02)	
South Asia	48 (23.88)	103 (51.24)	14 (6.97)	36 (17.91)	
Sub-Saharan Africa	7 (8.14)	43 (50.00)	9 (10.47)	27 (31.40)	
*Country/region where medical graduation was obtained*					
East Asia and Pacific	12 (28.57)	22 (52.38)	1 (2.38)	7 (16.67)	0.211
Europe and Central Asia	7 (16.28)	26 (60.47)	3 (6.98)	7 (16.28)	
North America, Latin America, and the Caribbean	10 (16.95)	35 (59.32)	4 (6.78)	10 (16.95)	
Middle East and North Africa	18 (16.36)	59 (53.64)	7 (6.36)	26 (23.64)	
South Asia	45 (25.00)	90 (50.00)	14 (7.78)	31 (17.22)	
Sub-Saharan Africa	7 (9.21)	39 (51.32)	7 (9.21)	23 (30.26)	
*Length of stay in Canada (years)*					
<3	8 (7.55)	52 (49.06)	11 (10.38)	35 (33.02)	<0.001
3–5	19 (15.20)	71 (56.80)	4 (3.20)	31 (24.80)	
6–10	38 (21.71)	98 (56.00)	17 (9.71)	22 (12.57)	
11–15	19 (36.54)	21 (40.38)	3 (5.77)	9 (17.31)	
>15	14 (18.92)	28 (37.84)	26 (35.14)	6 (8.11)	
*Years passed after graduation*					
<3	1 (5.26)	10 (52.63)	1 (5.26)	7 (36.84)	<0.001
3–5	5 (9.09)	29 (52.73)	1 (1.82)	20 (36.36)	
6–10	17 (13.82)	74 (60.16)	9 (7.32)	23 (18.70)	
11–15	16 (14.95)	64 (59.81)	9 (8.41)	18 (16.82)	
>15	59 (25.32)	97 (41.63)	41 (17.60)	36 (15.45)	
*Highest level of practice before coming to Canada*					
Medical student	11 (17.74)	35 (56.45)	3 (4.84)	13 (20.97)	<0.001
Resident	14 (17.95)	44 (56.41)	5 (6.41)	15 (19.23)	
Attending physician/hospital physician	21 (26.92)	34 (43.59)	4 (5.13)	19 (24.36)	
Consultant physician/specialist	18 (14.29)	52 (41.27)	35 (27.78)	21 (16.67)	
General practitioner/family physician	35 (18.23)	107 (55.73)	15 (7.81)	35 (18.23)	
Department head	0 (0.00)	5 (45.45)	5 (45.45)	1 (9.09)	
*Earnings (yearly)*					
≤$30,000	33 (12.31)	148 (55.22)	12 (4.48)	75 (27.99)	<0.001
$30,000–$39,999	15 (22.73)	30 (45.45)	6 (9.09)	15 (22.73)	
$40,000–$49,999	16 (21.62)	24 (32.43)	31 (41.89)	3 (4.05)	
$50,000–$59,999	13 (25.00)	33 (63.46)	3 (5.77)	3 (5.77)	
≥$60,000	22 (33.33)	36 (54.55)	7 (10.61)	1 (1.52)	

Note: Due to missing responses, some variable cell counts may not add up to total population number. * *p*-value from Pearson’s chi-squared test or Fisher’s exact test as appropriate.

## Data Availability

The datasets generated and analyzed during the current study are not publicly available due unavailability of the participants’ permission, privacy considerations, and ethical restrictions. Aggregated-level data might be available from the corresponding author on reasonable request after all the planned publications are completed.

## Supplementary Materials

The following supporting information can be downloaded at: https://www.mdpi.com/article/10.3390/healthcare10091705/s1. Table S1. Checklist for Reporting Results of Internet E-Surveys (CHERRIES).

## Abbreviations

CI: confidence interval; IMG: international medical graduate; IP: internet protocol; OR: odds ratio; USA: United States of America.
